# Regulation of Abiotic Stress Signalling by Arabidopsis C-Terminal Domain Phosphatase-Like 1 Requires Interaction with a K-Homology Domain-Containing Protein

**DOI:** 10.1371/journal.pone.0080509

**Published:** 2013-11-26

**Authors:** In Sil Jeong, Akihito Fukudome, Emre Aksoy, Woo Young Bang, Sewon Kim, Qingmei Guan, Jeong Dong Bahk, Kimberly A. May, William K. Russell, Jianhua Zhu, Hisashi Koiwa

**Affiliations:** 1 Department of Horticultural Sciences, Texas A&M University, College Station, Texas, United States of America; 2 Division of Applied Life Science (BK21 Program), Graduate School of Gyeongsang National University, Jinju, Gyeongsangnam-do, Korea; 3 Department of Plant Science and Landscape Architecture, University of Maryland, College Park, Maryland, United States of America; 4 Department of Chemistry, Texas A&M University, College Station, Texas, United States of America; Iwate University, Japan

## Abstract

*Arabidopsis thaliana* CARBOXYL-TERMINAL DOMAIN (CTD) PHOSPHATASE-LIKE 1 (CPL1) regulates plant transcriptional responses to diverse stress signals. Unlike typical CTD phosphatases, CPL1 contains two double-stranded (ds) RNA binding motifs (dsRBMs) at its C-terminus. Some dsRBMs can bind to dsRNA and/or other proteins, but the function of the CPL1 dsRBMs has remained obscure. Here, we report identification of REGULATOR OF CBF GENE EXPRESSION 3 (RCF3) as a CPL1-interacting protein. RCF3 co-purified with tandem-affinity-tagged CPL1 from cultured *Arabidopsis* cells and contains multiple K-homology (KH) domains, which were predicted to be important for binding to single-stranded DNA/RNA. Yeast two-hybrid, luciferase complementation imaging, and bimolecular fluorescence complementation analyses established that CPL1 and RCF3 strongly associate in vivo, an interaction mediated by the dsRBM1 of CPL1 and the KH3/KH4 domains of RCF3. Mapping of functional regions of CPL1 indicated that CPL1 *in vivo* function requires the dsRBM1, catalytic activity, and nuclear targeting of CPL1. Gene expression profiles of *rcf3* and *cpl1* mutants were similar during iron deficiency, but were distinct during the cold response. These results suggest that tethering CPL1 to RCF3 via dsRBM1 is part of the mechanism that confers specificity to CPL1-mediated transcriptional regulation.

## Introduction

Plants respond to environmental perturbations by rapid induction of suites of genes that promote plant adaptation to the altered environment. In *Arabidopsis thaliana*, CARBOXYL-TERMINAL DOMAIN (CTD) PHOSPHATASE-LIKE 1 (CPL1) regulates transcriptional responses to multiple environmental stresses including osmotic-stress/abscisic acid (ABA) and iron (Fe) deficiency stress [1], [2]. *cpl1* mutations cause hyperinduction of osmotic stress/ABA induced genes, such as *RESPONSIVE TO DEHYDRATION* (*RD*) and *COLD REGULATED* (*COR*), and show greater sensitivity to osmotic stress and ABA [2], [3]. Similarly, *cpl1* mutants overexpress Fe-deficiency-induced genes and exhibit a metal accumulation profile distinct from the wild-type profile [1], [4].

Sequence similarity and biochemical analyses suggest that CPL1 functions as a protein phosphatase that dephosphorylates the CTD of RNA polymerase II (pol II) [2], [5]. The CTD of Arabidopsis pol II consists of heptad repeats with the consensus sequence Y^1^S^2^P^3^T^4^S^5^P^6^S^7^ [6]. In animals and fungi, all residues except proline are reversibly phosphorylated and their phosphorylation status regulates various functions of pol II during transcription [7]. *Arabidopsis* CPL1 and its paralog CPL2 contain a conserved acid phosphatase motif at the N-terminal catalytic domain and C-terminal double-stranded (ds) RNA binding motifs (dsRBMs), and are able to specifically dephosphorylate the pol II CTD at Ser-5-PO_4_ [5], [8].

Many factors involved in transcription elongation, mRNA maturation and export, chromatin structure modification, and microRNA production have been identified as regulators of osmotic stress and ABA signalling in *Arabidopsis* [2], [3], [9]–[14], and are collectively referred as RNA metabolism proteins [15]. One such protein, *REGULATOR OF C-REPEAT BINDING FACTOR GENE EXPRESSION 3* (*RCF3*) [16], was identified as a negative regulator of cold responsive gene expression, with a mutant phenotype similar to *cpl1. RCF3* encodes a nuclear-localized K homology (KH) domain-containing protein. Proteins with KH domains are widely found in prokaryotes and eukaryotes, and are associated with transcriptional and translational regulation [17], [18]. A typical KH domain protein, such as heterogeneous nuclear ribonucleoprotein (hnRNP) K, is a multifunctional protein. For example, hnRNP K is one of the major pre-mRNA-binding proteins, and likely regulates nuclear metabolism of pre-mRNA [19]. hnRNP K can also bind to single-stranded DNA and regulate transcription [20], [21]. In *Arabidopsis*, 26 genes encode proteins with one or more KH domains [22], and genetic analysis of *FLOWERING LOCUS K* [23] and *HUA1 ENHANCER4* [24], as well as analysis of *RCF3*, has indicated the importance of KH domain proteins in plant growth and development. However, only limited information is available on how the KH domain functions in transcriptional regulation.

Here we report identification of RCF3 as a CPL1-interacting protein. RCF3 was identified in a tandem-affinity purified CPL1-containing complex by mass spectrometry. Independent binding analyses using yeast two-hybrid, luciferase complementation imaging (LuCI), and bimolecular fluorescence complementation (BiFC) assays established a specific interaction between CPL1 and RCF3. Moreover, genetic complementation of *cpl1-2* mutants using deletion variants of CPL1 suggested that the CPL1-RCF3 interaction is an integral part of in vivo CPL1 function. Comparison of gene expression profiles from *cpl1* and *rcf3* mutants suggested that CPL1 and RCF3 function in the same environmental responses, but each shows unique patterns of gene regulation, which overall suggests that RCF3 affects a subset of CPL1-regulated genes, perhaps including those in the negative feedback pathway in the osmotic stress signalling. These results indicate that the CPL1-RCF3 complex is functional and regulates abiotic stress signalling in plants.

## Materials and Methods

### Plant materials and growth conditions

The *Arabidopsis* (*Arabidopsis thaliana*) ecotype Col-0 was used in this study. *cpl1-6* and *rcf3-2* were described previously [1], [16]. *rcf3-2 cpl1-6* double mutant was prepared by genetic cross. For general growth, seeds were sown on medium containing half-strength Murashige and Skoog (MS) salts, 1% sucrose, and 0.8% agar. After stratification for 2 d at 4°C, the plates were kept in a growth incubator under long-day photoperiod (16 h light, 8 h darkness) at 25°C for 10 d.

### Stress treatments

Fe deficiency tests were performed as described [1]. Seeds were sown on basal medium containing one-quarter-strength (1/4 x) MS salts, 50 µM Fe-EDTA, 0.5% sucrose, and 1.5% agar. Fe deficiency was induced by transferring 7-d-old seedlings to basal medium without Fe-EDTA but containing 300 µM ferrozine [3-(2-pyridyl)-5,6-diphenyl-1,2,4-triazine sulfonate]. For testing cold stress, seeds were grown on basal medium for 7 days, and cold treated at 0°C for 24 h.

### Transgene constructs

The sequences of Entry clones for plant transformations are provided in Data S1. To express a tagged CPL1 in *Arabidopsis*, the *CPL1* coding sequence was placed upstream of 3xFLAG tag and SG-tag [25] of pEnSOFSGThsp. The resulting pEnSOCPL1FSGThsp (Data S1) was recombined with pMDC99 [26] using LR clonase (Life Technologies) to obtain pMDC99-SOCPL1FSGThsp. Gene expression cassettes for complementation and GFP-localization analyses were prepared based on pENTR-CPL1 containing the *CPL1* gene as an 8.4 kbp BlpI fragment of BAC clone F17L22. Subsequently, pENTRCPL1 derivatives were recombined with pBSVirHygGW [8]. These plasmids were introduced into *Agrobacterium tumefaciens* GV3101 [27] or GV3101 (pMP90RK) [28] and were used for transformation [2].

### Plant transformation and callus induction

*Arabidopsis* plants were grown under 16 hr light/8 hr dark at 23°C. Bolting stage plants were treated with Agrobacterium containing pMDC99-SOCPL1FSGThsp. Seeds from treated plants were germinated on media containing 1/4x MS salts, 0.7% agar, 25 µg/mg hygromycin B and 100 µg/ml Clavamox. Hygromycin-resistant seedlings were screened by immunoblot using anti-FLAG-HRP conjugate (see below). Positive plants were then cut into small pieces and cultured on callus induction media [29] to induce callus for cell culture.

For complementation of *cpl1-2*, a *cpl1-2 RD29a-luciferase* (*LUC*) line [3] was used for transformation and transformants were selected as described above.

### Tandem affinity purification (TAP)

TAP was performed as described [25] with slight modifications. Seven-day-old callus (total 40 g) was ground to a fine powder in liquid nitrogen and suspended in two volumes of CelLytic P protein extraction buffer (Sigma-Aldrich) supplemented with 1x Protease Inhibitor cocktail (Sigma-Aldrich). The extract was filtered through four layers of miracloth, and centrifuged at 15000 rpm for 15 min at 4°C. The cleared supernatant was mixed with 150–200 µl of IgG-Sepharose beads (GE Healthcare) and incubated at 4°C for 2 hr. After centrifugation at 1000 rpm for 1 min, IgG supernatant was discarded and the collected IgG beads were washed 3 times with 5 ml IgG washing buffer (10 mM Tris-HCl pH 8.0, 150 mM NaCl, 0.5% Triton X-100, 5% ethylene glycol) and then with 5 ml TEV cleavage buffer (25 mM Tris–HCl pH 8.0, 150 mM NaCl, 0.1% NP-40, 0.5 mM EDTA, 1 mM DTT) at 4°C for 10 min. The bound complexes were eluted by cleavage with tobacco etch virus (TEV) protease in TEV cleavage buffer for 16 hr at 4°C. The eluate was then incubated with 70 µl of streptavidin Sepharose beads (GE Healthcare) for 4 h at 4°C. The beads were washed 3 times with 1 ml of TEV cleavage buffer and then the protein complexes were eluted with elution buffer (20 mM Desthiobiotin, 0.1% Triton X-100 in TEV cleavage buffer. The proteins in the eluate were concentrated with centricon YM-10 (Millipore) and loaded onto a 10% polyacrylamide gel for SDS-PAGE.

### In-gel proteolytic digestion and MALDI-TOF

For protein identification by mass spectrometry (MS), the protein bands of interest were manually excised (approximately 2 mm strips) and placed in microcentrifuge tubes for in-gel digestion as previously described [30], [31]. Briefly, the isolated gel plugs were subjected to proteolysis by proteome grade trypsin (Sigma Aldrich) at pH 8, 37°C, for at least 4 hours before analysis by mass spectrometry. The peptide mix was desalted using C18 ZipTips (Millipore) and 1 µl of this solution was combined with 1 µl of a 3 mg/ml α-cyano-4-hydroxycinnamic acid (60% acetonitrile, 1 mM ammonium diphosphate) and spotted onto MALDI targets. All MALDI-MS experiments were performed using a 4800 MALDI-TOF/TOF (Applied Biosystems). The MS data were acquired using the reflectron detector in positive mode (700–4500 Da, 1900 Da focus mass) using 800 laser shots (40 shots per sub-spectrum) with internal calibration. Collision induced dissociation tandem MS spectra were acquired using 10–20% greater laser power than the MS spectra acquisition using 2 kV of collision energy. All MS and MS/MS data were searched against the UniProt protein sequence database using the GPS Explorer (Applied Biosystems) software [32]. The database search parameters used for Mascot (Matrix Science, London, UK) were the following: precursor mass tolerance: 50 ppm, taxonomy: all entries and *Arabidopsis thaliana*, enzyme: trypsin, missed cleavages: 1, and variable modifications: oxidation (M).

### Luciferase Complementation Imaging (LuCI) assay

LuCI was performed as described [33]. CPL1 and RCF3 fragments were cloned in pDONRzeo (Life Technologies) by Gateway BP reaction and then transferred into pDEST-NLUC^GW^ or pDEST-CLUC^GW^ [33] by Gateway LR reaction (Life Technologies). Resulting NLUC/CLUC constructs and a 35S-P19 construct (provided by Dr. Baulcomb) were introduced into *Agrobacterium tumefaciens* GV3101 cells [27].

To test interactions, GV3101 cells carrying the various NLUC/CLUC constructs were prepared as follows. Cells grown on solid LB medium supplemented with 50 µg/ml kanamycin were inoculated in 10 ml of liquid LB kanamycin medium. After 20 h incubation, cells were harvested by centrifugation at 4000 rpm for 10 min and re-suspended in fresh activation medium containing 10 mM MES/KOH (pH 5.6), 10 mM MgCl_2_ and 150 µM acetosyringone. Cell suspensions were mixed to achieve a final OD_600_ of 0.4 for NLUC/CLUC constructs and 0.15 for the P19 helper strain, respectively. The 100 µl of NLUC, CLUC and P19 cell suspension mixtures were infiltrated into leaves of 4- to 7-week-old *Nicotiana benthamiana* plants. Luminescence images were taken 3 d after infiltration. Leaves were infiltrated with luciferin solution (10 mM MES/KOH, pH 5.6, 10 mM MgCl_2_ and 100 µM luciferin) and images were acquired using an electron multiplying charge coupled device camera (EMCCD, Cascade II, Roper Scientific) and processed by WinView software (Roper Scientific).

### Bimolecular Fluorescence Complementation (BiFC)

cDNA fragments encoding a full-length RCF3 and a C-terminal 327-amino-acid fragment of CPL1 (CPL1^640–967^) were cloned into BiFC vectors [34] to produce pRCF3-nYFP and pCPL1^640–967^-cYFP. CPL1^640–967^ contains both dsRBMs and nuclear localization signal of CPL1 and its translation starts with an internal Met^640^ of CPL1. For negative controls, pMYB75-nYFP and pMYB75-cYFP were prepared using an unrelated transcription factor, *Arabidopsis* MYB75. The transformation DNA mixtures contained the indicated combinations of 10 µg of each DNA preparation. Polyethylene glycol-mediated transformation of *Arabidopsis* protoplasts were performed as described [5].

### Yeast two-hybrid assays

For the yeast two-hybrid analysis, *CPL1*, *CPL2* and *RCF3* fragments were amplified by PCR and cloned into pDONRzeo by the Gateway BP reaction. Gateway compatible two-hybrid vectors, pBUTE^GW^ and pGAD^GW^, were prepared by inserting Gateway cassette A (Life Technologies) into the SmaI site of pBute [35] or pGAD.c1 [36], and were used to clone *CPL1* and *RCF3* fragments by Gateway LR reactions. Lithium acetate-mediated transformation of yeast strain PJ69-4A was performed as described [37]. After transformation, yeast were plated on synthetic dropout media (SD) composed of nitrogen base, 2% glucose and a dropout supplement without uracil and leucine (-UL) and incubated at 28°C for 48 hr. 2×10^5^ cells of colonies growing on SD/-UL and their diluted cells (2×10^4^ cells) were transferred onto SD composed of nitrogen base, 2% glucose, a dropout supplement without uracil, leucine, histidine and adenine (-ULHA) and 40 µg/ml 5-bromo-4-chloro-3-indolyl-α-D-galactopyranoside (X-α-gal, Goldbio) and incubated at 28°C for 48 h. pGAD-RanBPM was used as positive control [38].

### Subcellular localization analysis

*CPL1* cDNA encoding the C-terminal region was introduced into pEnSOTG [5] to prepare GFP-CPL1 expression plasmids. Ten micrograms of resulting plasmid DNA was introduced into *Arabidopsis* protoplasts by polyethylene glycol-mediated transformation as described [5]. DsRed protein fused to the SV40 nuclear localization signal (NLS) sequence was used as a positive control for nuclear localization [39]. Transformed protoplasts were incubated at 22°C in the dark. Expression of the fusion protein was observed with an Olympus AX-70 fluorescence microscope 2 and 3 days after transformation, and the images were captured with a cooled charge-coupled device camera (Olympus DP-70). The filter sets used were XF116-2 (exciter, 475AF20; dichroic, 500DRLP; emitter, 510AF23) and XF33 (exciter, 535DF35; dichroic, 570DRLP; emitter, 605DF50) (Omega, Inc., Brattleboro, VT, USA) for GFP and DsRed, respectively.

### Luciferase assay

Growth and cold treatment of wild type, *cpl1-2* mutants and complemented lines were performed as described previously [2]. For luciferase image acquisition, plants were sprayed with luciferin solution (0.01% TritonX-100, 1 mM luciferin) and were kept in the dark for 5 min before image acquisition and processing, as described above.

### Total protein extraction and immunoblot analysis

Two-week-old transgenic plants were homogenized in extraction buffer [50 mM Tris–HCl pH 8.0, 1 mM EDTA, 12.5% glycerol, 1 mM dithiothreitol, 1 mM phenylmethylsulfonyl fluoride, 1x complete protease inhibitor cocktail (Sigma)]. After centrifugation at 12,000 rpm for 10 min, protein concentration in the supernatant was determined by Bradford reagent assay. 20 µg of total protein extracts were separated on a 7.5% SDS-PAGE gel, and electroblotted onto nitrocellulose membranes. Subsequently, the membranes were blocked with 6% skim milk in TBS buffer (20 mM Tris-Cl pH 7.5, 0.8% NaCl) + 0.05% Tween 20 (TTBS), washed 4 times with TTBS for 5 min, and probed with anti-FLAG-HRP conjugate (1:200,000, Sigma) in 3% milk in TTBS for overnight at 4°C. The membrane was washed 4 times with TTBS for 5 min before developing. CPL1-FLAG proteins were detected using a Supersignal West Femto chemiluminescence detection reagent (Thermo Scientific) and EMCCD camera.

### Confocal laser scanning microscopy

One-week-old root tissues grown on media containing 1/2x MS salts and 1% sucrose were stained for 10 sec in an aqueous solution containing 10 µg/ml propidium iodide (Sigma-Aldrich), which stains the cell walls in living cells. The root were then rinsed and mounted in distilled water under a coverslip. To observe fluorescence, a FluoView FV1000 confocal microscope (Olympus) was used. Excitation and emission of GFP were at 488 and 510–540 nm, respectively, and the excitation and emission of propidium iodide were at 543 and 587–625 nm, respectively.

### Reverse transcription-quantitative PCR

Total RNA extraction, reverse transcription and quantitative PCR analysis was performed as described [1]. Primer sequences were as described [1].

### Accession numbers

Sequence data from this article can be found in the Arabidopsis genome initiatives under the accession numbers *CPL1*, At4g21670; *CPL2*, At5g01270; *RCF3*, At5g53060; MYB75, At1g56650; *CBF2*, At4g25470; *CBF3*, At4g25480; *RD29*a, At5g52310; *IRT1*, At4g196900; *FRO2*, At1g015800; *FIT*, At2g28160; *bHLH38*, At3g569700; *bHLH39*, At3g569800; *bHLH100*, At2g412400; *bHLH101*, At5g041500; *LEA (LATE EMBRYOGENESYS ABUNDANT)*, At3g15670; *LEA18*, At2g353000; *LEA4-5*, At5g067600; *ABAR (ABA-RESPONSIVE PROTEIN-RELATED)*, At3g024800; *RAB18 (RESPONSIVE TO ABA)*, At5g664000; USP (*UNIVERSAL STRESS PROTEIN*), At3g584500; *COR47* (*COLD-REGULATED 47*), At1g204400, and in the EMBL/GenBank data libraries under accession numbers hnRNP K, P61978; PCBP3, AAH12061.

## Results

### The CPL1 C-terminal region interacts with RCF3

**Figure 1:**
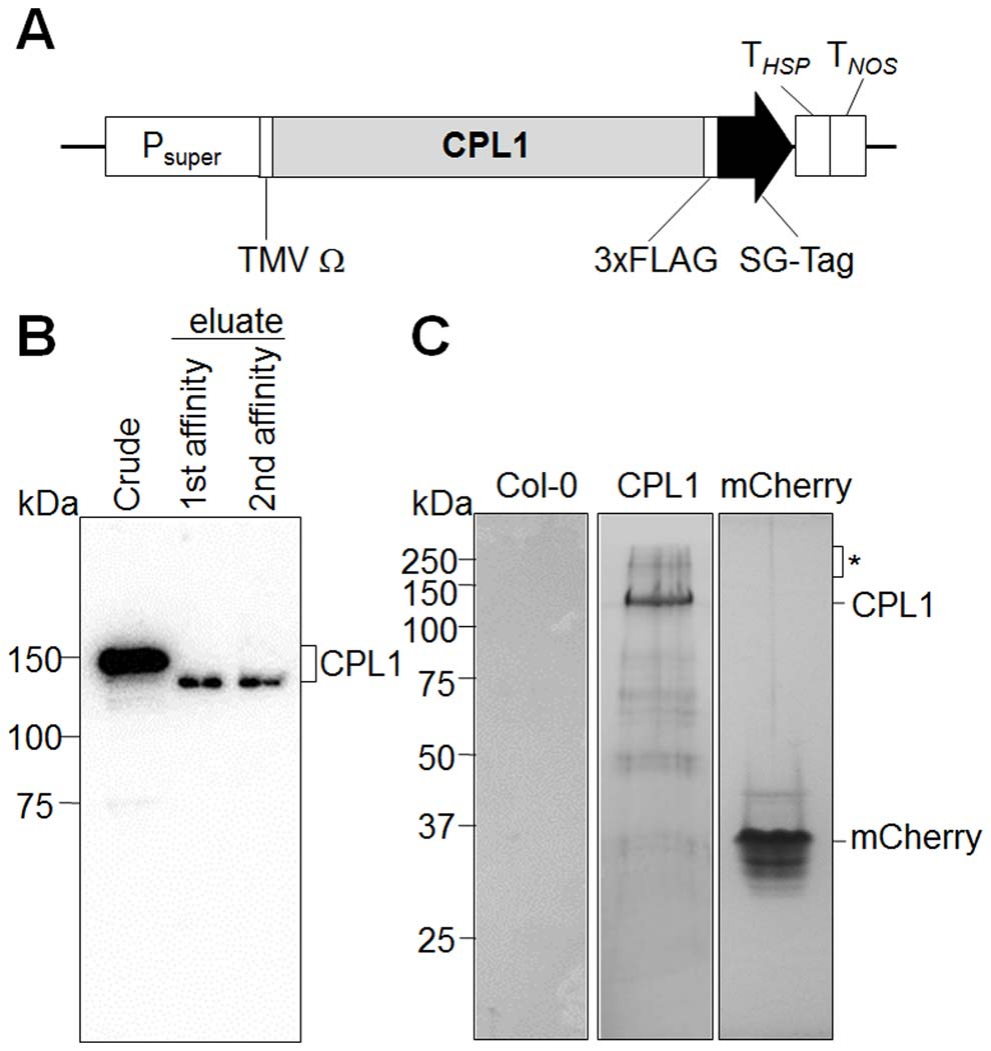
Isolation of CPL1 complex components by tandem affinity purification (TAP). (A) Schematic representation of the expression cassette for FSG-tagged CPL1. The expression of CPL1-FSG is controlled by super promoter (Psuper), the 5′-leader sequence of tobacco mosaic virus (TMV Ω), and tandem terminators (HSP18 and NOS terminators). The C-terminus of CPL1 was fused to 3xFLAG tag followed by the SG-TAP tag. (B) Purification of the CPL1 complex from crude extracts of transformed calli. Total extracts of transformed calli and eluate after each affinity purification step were separated on a 7.5% SDS-PAGE gel, and tagged-CPL1 was visualized by immunoblot using anti-FLAG-HRP conjugate. (C) Protein profile of CPL1 complex after tandem affinity purification. Final eluate was separated on 10% SDS-PAGE gel and stained by Coomassie Brilliant Blue R-250. Asterisk indicates the position of high-molecular-weight fraction analyzed in this study. Control tandem-affinity eluates from untransformed Col-0 cells and mCherry-FSG cells are shown for comparison.

We have previously identified several CPL1-interacting proteins by yeast two-hybrid screening [37]. As a complementary strategy, we conducted new searches for *in planta* CPL1-associating proteins using a proteomics-based approach. CPL1 fused to [3xFLAG]-[Streptavidin-binding peptide]-[Protein G] tandem-affinity-purification tag (FSG-tag) was expressed in *Arabidopsis* cell culture; these cells produced an anti-FLAG immuno-positive band of ca. 145 kDa corresponding to CPL1-FSG peptide (Figure 1A). A tandem affinity purification (TAP) procedure resulted in recovery of a 120 kDa immuno-positive peptide due to the cleavage of protein G domains during purification. The TAP-purified CPL1 fraction was resolved by SDS-PAGE, which produced a predominant band corresponding to CPL1, as detected by immunoblot (Figure 1B). The control TAP fraction from untransformed cells did not yield notable bands (Figure 1C, left), and one from cells expressing TAP-tagged mCherry showed a predominant band of mCherry and minor low-molecular weight bands (Figure 1C, right), which did not overlap with the bands observed in the affinity-purified CPL1 fraction (Figure 1C, middle). Interestingly, the purified CPL1 fraction contained high-molecular weight proteins that migrated at >200 kDa. Preliminary tandem MALDI-TOF/TOF Mass Spectrometry analysis of this high molecular weight fraction identified CPL1 (Mascot Protein Score 538, Confidential Interval 100%) and RCF3 encoded by At5g53060 (Mascot Protein Score 68, Confidential Interval 99.1%, Figure S1). RCF3 was not identified as a CPL1-associating protein in the previous two-hybrid screen. Most of the other visible bands corresponded to degradation products of CPL1. A full profile of proteins co-purified with CPL1 will be described elsewhere.

**Figure 2:**
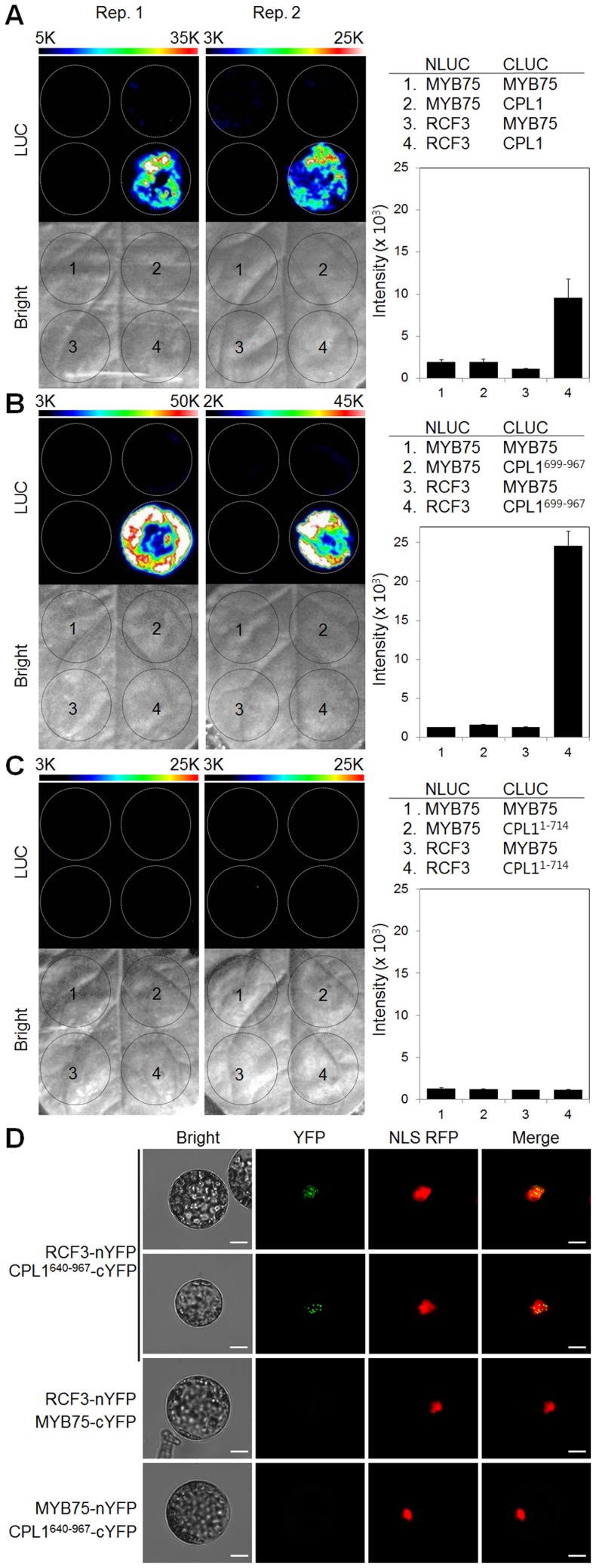
CPL1 and RCF3 specifically interact in vivo. Luminescence (LUC) and bright-field images (Bright) of LuCI assays using CLUC fused with CPL1 (A), CPL1^699–967^ (B) and CPL1^1–714^ (C) fragments and NLUC fused with RCF3 are shown. Mixtures of Agrobacterium cell suspensions (100 µl) containing LuCI expression cassettes were infiltrated into 4-7-week-old *Nicotiana benthamiana* leaves. LUC images were obtained 3 days after infiltration. The dotted circles indicate the areas used for Agrobacterium infiltrations. The graphs represent luminescence intensities inside of the each circle. Bars indicate standard errors of the mean from the two replicates (Rep. 1 and Rep. 2). (D) BiFC assay of CPL1^640–967^ and RCF3 interactions in *Arabidopsis* protoplasts. Plasmids encoding expression cassettes for CPL1^640–967^ and RCF3 fused with C- or N terminal fragments of YFP (cYFP or nYFP), respectively, were transfected into *Arabidopsis* protoplasts. Reconstituted YFP fluorescence was monitored using standard FITC and rhodamine filter sets one day after transformation. MYB75 was used as a negative control. NLS-RFP was used as a positive control for nuclear localization. Yellow signals on merged images indicate co-localization of YFP and RFP proteins. Scale bars indicate 10 µm.

The protein-protein interaction between CPL1 and RCF3 in vivo was confirmed by luciferase complementation image (LuCI) analysis [33]. In this analysis, CPL1 and RCF3 were transiently expressed as fusion proteins with an N-terminal 416-amino-acid or C-terminal 153-amino-acid fragment of firefly luciferase (NLUC or CLUC). Co-expression of fusion proteins that form a protein complex brings the two halves of LUC in close proximity, and allows reconstitution of an active LUC. NLUC-RCF3 transiently coexpressed with CLUC-CPL1 or the truncated CLUC-CPL1^699–967^ in *Nicotiana benthamiana* leaves reconstituted LUC activity (Figure 2A and B). By contrast, NLUC-RCF3 coexpressed with CLUC-CPL1^1–714^ (Figure 2C), and negative control combinations using LUC fragments fused to an unrelated nuclear-localized control protein (transcription factor MYB75) did not produce luciferase activity. Together, these results establish that CPL1 and RCF3 form a complex in vivo, via the C-terminal region of CPL1, which contains dsRBMs.

We used BiFC to determine the subcellular location of the CPL1-RCF3 complex. For this purpose, fusion proteins, i.e., RCF3-nYFP and CPL1^640–967^-cYFP were co-expressed in *Arabidopsis* mesophyll protoplasts. Because of the large size of full-length CPL1, only the C-terminal fragment, starting with an internal Met codon and containing the dsRBMs, was used. The co-expression of RCF3-nYFP and CPL1^640–967^-cYFP, but not other control combinations, produced fluorescent signal (Figure 2D), confirming the specific interaction between CPL1 and RCF3. The fluorescent signals from the CPL1-RCF3 complex localized to nuclei, consistent with the location of individual proteins reported previously [5], [16]. However, unlike the rather uniform nucleoplasmic fluorescence produced from individually expressed CPL1-GFP or RCF3-GFP proteins [5], [16], the fluorescent signals produced from the CPL1-RCF3 complex formed speckles in the nuclei. The observed pattern did not vary among the individual cells with varying fluorescence intensities, suggesting that complex formation, rather than protein expression level, was important for confining proteins to speckles. Together, these results establish that CPL1 and RCF3 specifically interact in Arabidopsis nuclei.

### RCF3 encodes a protein with five canonical KH-domains

A search against the Conserved Domain Database [40], [41] identified five KH domains in RCF3 (Figure S2). All of these are eukaryotic type I KH domains and are homologous to those found in the hnRNP K and poly-r(C)-binding protein (PCBP) family proteins. Alignment of individual RCF3 KH domains with human hnRNP K and PCBP3 KH domains highlighted several features of the RCF3 KH domains. Overall, RCF3 KH domains showed a higher level of sequence conservation in the KH minimal motif region, the β3 region, and the α3 region. KH2 and KH4 contained a short “variable loop”, which is often observed in type I KH domains. Interestingly, KH1 and KH3 contained another loop sequence between the β3 and the α3 regions. Sequence comparisons of multiple KH domain proteins suggested that an insertion between the β3 and the α3 regions is frequently observed in plant KH domains (variable loop 2), but rarely found in KH domains of other organisms. Notably, the KH2 sequence contains an extra residue between conserved glycines, deviating from the highly conserved GXXG motif consensus sequence. In addition, KH5 lacks both glycines in the GXXG region.

### Peptide regions important for the CPL1-RCF3 interaction

**Figure 3:**
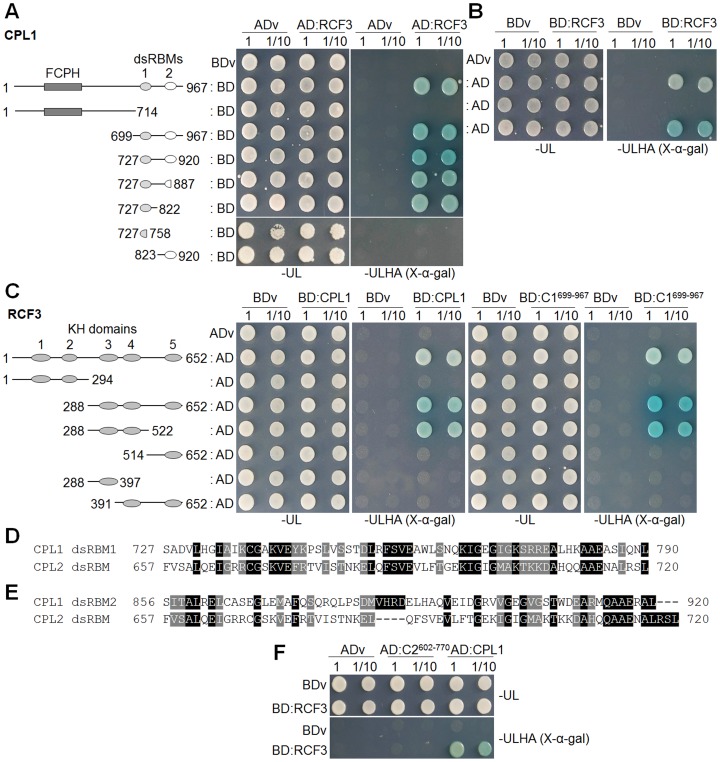
Yeast two-hybrid analysis of the interaction of CPL1 or CPL2 dsRBM with RCF3. (A) Growth of PJ69-4A co-expressing GAL4 DNA binding domain (BD) fused with various CPL1 peptide fragments and GAL4 activation domain (AD) fused with RCF3 (AD:RCF3). FCPH, Fcp1 homology domain; dsRBM, double-stranded RNA-binding motif. (B) Growth of PJ69-4A co-transformed with GAL4-AD fused with various CPL1 fragments and GAL4-BD fused with RCF3 (BD:RCF3). (C) Growth of PJ69-4A co-expressing GAL4-BD fused with CPL1 (BD:CPL1) or CPL1^699–967^ (BD:C1D) and GAL4-AD fused with various RCF3 fragments. Numbered ovals represent KH domains. Alignment of CPL2 dsRBM with CPL1 dsRBM1 (D) or dsRBM2 (E) using ClustalW. (F) Growth of PJ69-4A co-transformed with GAL4-BD fused with RCF3 (BD:RCF3) and GAL4-AD fused with CPL2 dsRBM (AD:C2^602–770^). GAL4-AD fused with full-length CPL1 (AD:C1F) was used as positive control. Cells were grown on synthetic dropout (SD) media without uracil and leucine (-UL) or SD medium without uracil, leucine, histidine and adenine (-ULHA) supplemented with 40 µg/ml X-α-gal. 2x10^5^ cells were used for (1) and diluted 10-fold for (1/10). Photographs were taken after incubation at 28°C for 48 hours. ADv and BDv indicate vector controls.

To map the region responsible for the CPL1 and RCF3 interaction, yeast two-hybrid analyses using fragments of CPL1 and RCF3 were performed. First, the reliability of yeast two-hybrid assays for examination of the CPL1-RCF3 interaction was assessed using full-length CPL1 and RCF3 proteins. Histidine autotrophy and α-galactosidase activity of the host cells, indicative of strong interaction of test proteins, were detected either when CPL1 was fused to GAL4 DNA-binding domain and RCF3 was fused to GAL4 activation domain (Figure 3A), or vice versa (Figure 3B). Furthermore, the CPL1 C-terminal region (CPL1^699–967^) was sufficient to interact with RCF3 (Figure 3A, B), consistent with the data obtained *in planta*. These results indicate that the yeast two-hybrid assay successfully reproduces the CPL1-RCF3 interaction observed *in planta*.

Subsequent analyses were conducted with serial deletion constructs encoding truncated CPL1 and RCF3 fragments. RCF3 fragments containing KH3 and KH4 interact with both full-length CPL1 and CPL1^699–967^ (Figure 3C). However, fragments containing the KH3 or KH4 domain separately or fragments lacking these domains failed to interact with CPL1, establishing that KH3 and KH4 are both required for interaction with CPL1. For CPL1, fragments containing the dsRBM1 could interact with RCF3 but fragments lacking dsRBM1 could not (Figure 3A), suggesting that dsRBM1 functions as the sole binding site for RCF3.

CPL2 is a paralog of CPL1 and contains a single dsRBM, which shows higher sequence similarity to CPL1 dsRBM1 than to dsRBM2 (Figure 3D and E). The *cpl1 cpl2* double mutant is pollen lethal, indicating that CPL1 and CPL2 share an essential function in plant development [5]. To test if RCF3 functions in a common pathway shared by CPL1 and CPL2, the interaction between CPL2 dsRBM and RCF3 was tested by yeast two-hybrid analysis. However, CPL2 and RCF3 did not interact (Figure 3F, Figure S3), suggesting the association with RCF3 is unique to CPL1 dsRBM1.

### Catalytic domain and the first dsRBM are essential for CPL1 function in vivo

**Figure 4:**
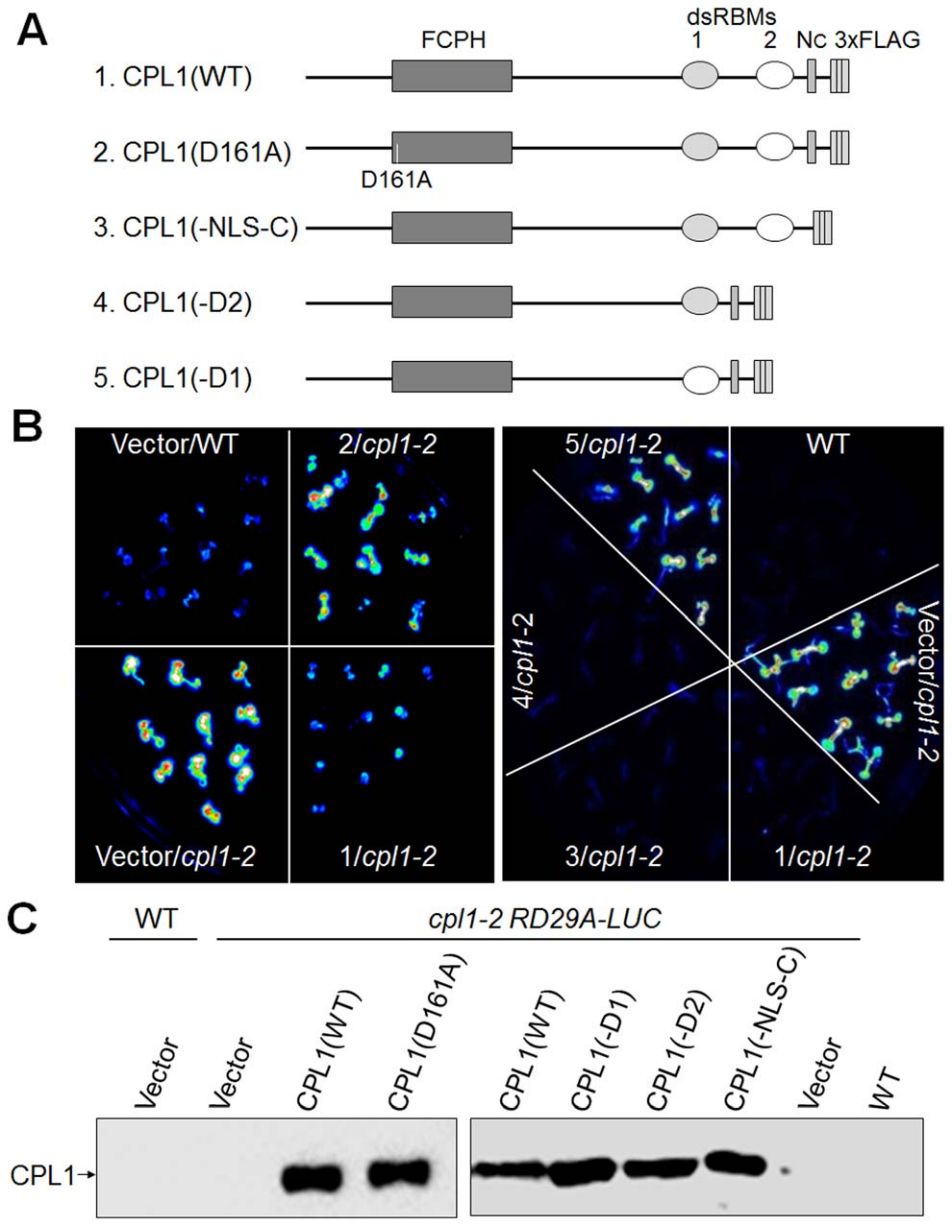
Functional analysis of CPL1 variants *in planta*. (A) Schematic representation of the domain structures of wild type and variant CPL1. FCPH, Fcp1 homology domain; dsRBM, double-stranded RNA-binding motif; N_C_, C-terminal NLS; 3xFLAG, 3xFLAG-tag; D161A, Asp to Ala amino acid replacement at catalytic motif. (B) Luminescence images visualizing *RD29A-LUC* reporter gene expression in transgenic lines. Wild type (WT) and *cpl1-2* plants were transformed with CPL1 variants shown in (A). Two-week-old plants were cold (0°C) treated for 48 h and subjected to LUC imaging. (C) Expression of CPL1-FLAG variants in transgenic lines used in (B). Total proteins were extracted from 2-week-old transgenic plants and 20 µg of protein were separated on 7.5% SDS-PAGE gels. Immunoblots were detected using *anti-FLAG*-HRP conjugate.

To determine the functional significance of the CPL1-RCF3 interaction, CPL1 variants lacking different domains were genetically tested for complementation of *cpl1-2* (formerly *fry2-1*) mutants with the *RD29a-LUC* reporter gene (*cpl1-2 RD29a-LUC*). In this reporter line, loss of function in *CPL1* results in characteristic hyperinduction of *LUC* when plants are exposed to osmotic stress, such as cold treatment [3]. The *cpl1-2* loss-of-function mutation has a disrupted splice site upstream of dsRBM1 and produces transcripts encoding a truncated protein [3], [5]. The resulting mutant phenotype suggests the importance of the dsRBMs in CPL1 *in vivo* function [2], [5]. Genomic fragments containing C-terminally 3xFLAG-tagged CPL1 with various mutations were prepared; these *CPL1-FLAG* constructs contain the native *CPL1* promoter, all exons and introns, and the *CPL1* terminator sequence, to produce a fusion protein regulated similarly to the native CPL1 (Data S1). The variants prepared include *CPL1(D161A)-FLAG*, in which the catalytic domain contains the Asp to Ala mutation in a highly conserved phosphatase motif, D^161^XDXT, and *CPL1* variants containing deletions at the 1st or the 2nd dsRBM (Figure 4A). In addition, to determine the significance of nuclear localization of CPL1, a *CPL1* variant lacking the C-terminal NLS was prepared. The NLS was previously determined to be located at the C-terminus (amino acid 945–967) of CPL1. Further mapping of the NLS in this region identified a 5-amino-acid motif (KRLKP: NLS-C) that was sufficient to target a GFP fusion protein to nuclei in protoplast assays (Figure S4). In the *CPL1(-NLS-C)-FLAG* construct, a 5-amino-acid deletion was introduced to remove the NLS-C sequence (Figure 4A).

These *CPL1-FLAG* constructs were introduced into *cpl1-2 RD29a-LUC*, and their function was determined based on the level of cold-induced expression of the reporter gene (Figure 4B). As expected, introduction of wild type *CPL1-FLAG* but not the catalytic domain variant *CPL1(D161A)-FLAG* into the *cpl1-2 RD29a-LUC* plant reverted the high *RD29a-LUC* expression level of the mutant down to the level of the wild-type *RD29a-LUC* line. Importantly, *CPL1(-dsRBM1)-FLAG* failed to rescue the *RD29a-LUC* hyper-expression of *cpl1-2*, whereas *CPL1(-dsRBM2)-FLAG* could. Expression of these *CPL1* variant proteins was confirmed by anti-FLAG immunoblot (Figure 4C). This indicates that catalytic activity and dsRBM1, but not dsRBM2, are essential for the in vivo function of CPL1. Since the deletion of dsRBM1, which disrupts the CPL1-RCF3 interaction, is as detrimental as a mutation that disrupts catalytic activity, formation of the CPL1-RCF3 complex via dsRBM1 is likely essential for CPL1 to regulate osmotic stress signalling.

### CPL1 contains redundant nuclear localization signals that are essential for its in vivo function

**Figure 5:**
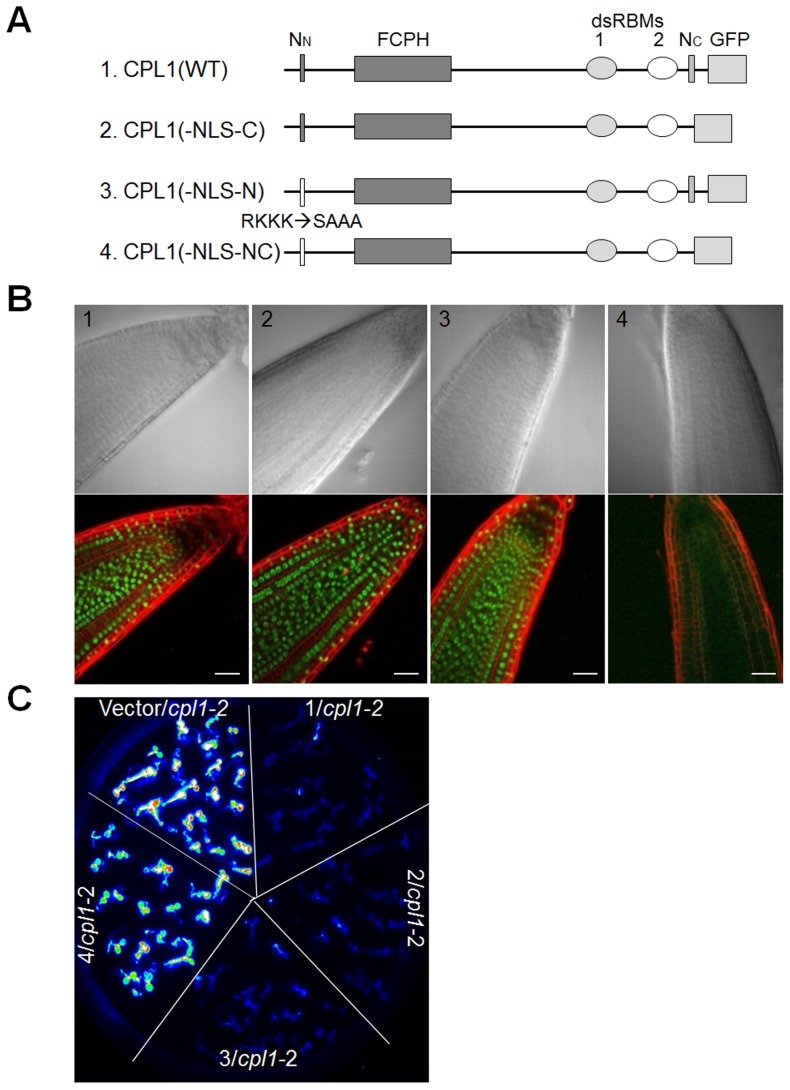
Nuclear targeting is essential for CPL1 function. (A) Schematic representation of the domain structures of wild type and variant CPL1 used for the analyses. FCPH, Fcp1 homology domain; dsRBM, double-stranded RNA-binding motif; N_N_, N-terminal NLS-like sequence; N_C_, C-terminal NLS; GFP, green fluorescent protein. RKKK -> SAAA, amino acid replacements for N-terminal NLS-like sequence. (B) Bright field (top) and fluorescence (bottom) images of *cpl1-2* plants expressing CPL1-GFP variants shown in (A). Green and red signals correspond to location of CPL1-GFP and cell walls stained with propidium iodide, respectively. Bars indicate 20 µm. (C) Luminescence images visualizing *RD29A-LUC* reporter gene expression in transgenic lines used in (B). Two-week-old plants were cold (0°C) treated for 48 h and subjected to LUC imaging analysis.

Surprisingly, *CPL1(-NLS-C)-FLAG* also effectively rescued the *cpl1-2 RD29a-LUC* phenotype (Figure 4B). This implies either that CPL1 functions outside of nuclei or that CPL1(-NLS-C)-FLAG localizes in nuclei due to a presence of an additional NLS. The second possibility was likely because another NLS-like sequence (RKKKQR: NLS-N) was found at the N-terminal region of CPL1 (amino acid 38–43) (Figure 5A). Roles of NLS-N/C sequences in subcellular localization and function of CPL1 were tested using *CPL1-GFP* fusion constructs, which were prepared by replacing the 3xFLAG-tag sequence of CPL1-FLAG with a GFP open reading frame. As shown in Figure 5B and C, wild type CPL1-GFP not only localized to nuclei, but also complemented the *cpl1-2 RD29a-LUC* phenotype. Interestingly, singly mutating either NLS-N or NLS-C sequence did not alter the nuclear targeting of CPL1-GFP and the ability to complement *cpl1-2 RD29a-LUC*, indicating these mutations do not individually interfere with CPL1 function. However, when NLS-N and NLS-C were mutated simultaneously, CPL1(-NLS-NC)-GFP fluorescence was no longer confined to nuclei and diffuse intracellular fluorescence was observed (Figure 5B). Furthermore, *CPL1(-NLS-NC)-GFP* was no longer able to complement *cpl1-2 RD29a-LUC* (Figure 5C). Together, these results establish that CPL1 has two redundant NLS, and nuclear localization is essential for *in vivo* function of CPL1.

### CPL1 and RCF3 Function in Overlapping Abiotic Stress Responses

**Figure 6:**
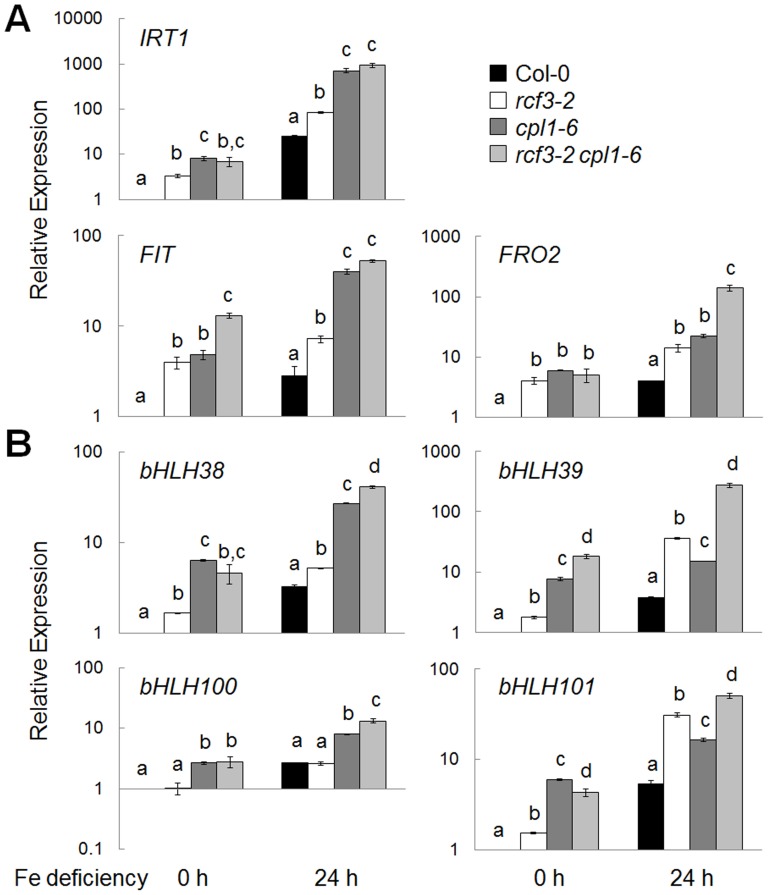
Expression levels of Fe-regulated genes in the roots of *rcf3-2, cpl1-6, rcf3-2 cpl1-6* and Col-0 under Fe deficiency. (A) Expression levels of FIT-dependent pathway genes. (B) Expression levels of FIT-independent pathway genes. Plants were grown on basal medium for 7 days, and then transferred to Fe-deficient basal medium containing 300 µM ferrozine. Root samples were collected at the time of transfer (0), or 24 h after the transfer. The presented expression levels (relative to untreated Col-0 samples) are mean values of two biological replicates analyzed in duplicates. Bars indicate standard errors of the mean (SEM) of biological replicates. Different letters show significant differences between genotypes under Fe+ and Fe- conditions (*p*<0.05, one-way ANOVA followed by Tukey's HSD post hoc test).

**Figure 7:**
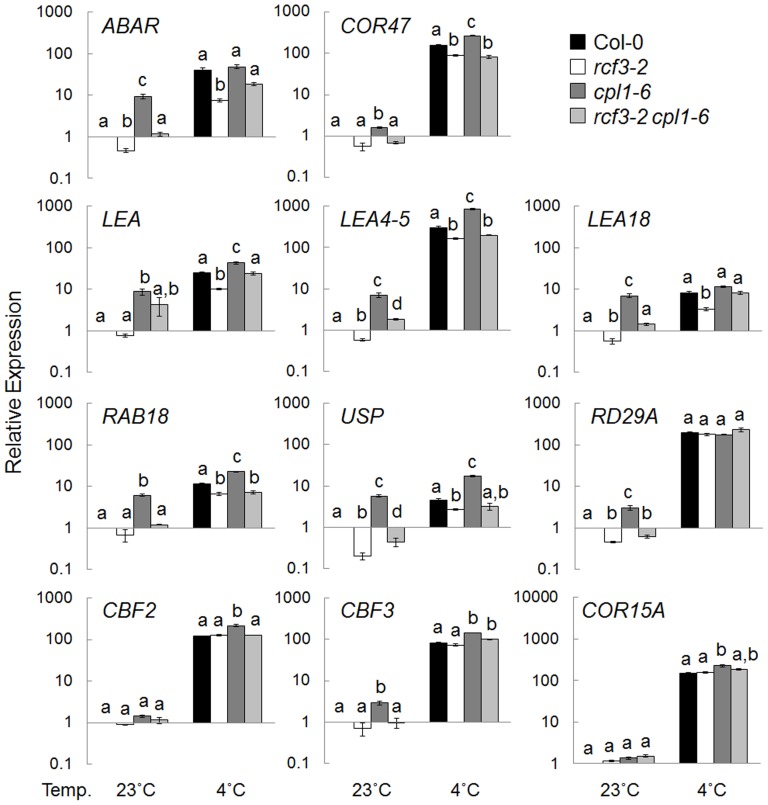
Expression levels of osmotic-stress regulated genes in *rcf3-2, cpl1-6, rcf3-2 cpl1-6*, and Col-0 seedlings. Plants were grown on basal medium for 7 days at 23°C, and then exposed to cold treatment (0°C, 24 h). The presented expression levels (relative to untreated Col-0 samples) are mean values of two biological replicates analyzed in duplicates. Bars indicate standard errors of the mean (SEM) of biological replicates. Different letters show significant differences between genotypes under the same conditions (*p*<0.05, one-way ANOVA followed by Tukey's HSD post hoc test).

Genetic interaction between *CPL1* and *RCF3* was analyzed using *cpl1, rcf3* single mutants and *cpl1 rcf3* double mutant. For this purpose, we used *cpl1-6* and *rcf3-2*, previously characterized T-DNA insertion mutants in Col-0 background. As molecular markers for stress responses, two classes of *CUTs* (*cpl1-UP Transcripts*) that represent various osmotic stress (cold, salinity, etc)-regulated (group I) and Fe-deficiency stress-regulated (group II) genes were used. Similar to the *cpl1*, the *rcf3-2* mutant responded to Fe deficiency by up-regulating group II *CUT*s that are involved in regulation of Fe acquisition (Figure 6) [1], [42], [43]. The increased expression was more evident with genes that are targets of FER-LIKE FE DEFICIENCY-INDUCED TRANSCRIPTION FACTOR (FIT), i.e., *IRON REGULATED TRANSPORTER 1* (*IRT1*) and *FERRIC REDUCTION OXIDASE 2* (*FRO2*) as well as *FIT* itself [44]. However, genes in the FIT-independent pathway [45] were not strongly upregulated by *rcf3*, except bHLH101, another Fe-response determinant [46]. In *rcf3-2 cpl1-6* double mutant, most of group II *CUTs* showed similar level to *cpl1-6*, suggesting RCF3 may function through CPL1. However, we observed additive effect of *cpl1-6* and *rcf3-2* for *FRO2* and *bHLH39* expression pattern. By contrast, the cold-induction of group I *CUTs* expression was impaired in *rcf3-2* (Figure 7). Out of 10 genes tested, cold-induced expression levels of 7 *CUT*s were significantly affected (*p*<0.05) by *rcf3-2* (Figure 7). While *cpl1-6* single mutant showed hyperinduction of the *CUTs* expression, *rcf3-2* strongly suppressed group I *CUT* expression in the *rcf3-2 cpl1-6* double mutant. Interestingly, the expression of transcription factors that regulate the expression of these protective genes, i.e., *C-REPEAT BINDING FACTOR (CBF) 2* and *CBF3* [47]–[49], was not affected by *rcf3-2* (Figure 7), but their hyper-inductions in *cpl1-6* were suppressed in *rcf3-2 cpl1-6*. These results suggest that during the cold response, CPL1 functions through RCF3. Overall, CPL1 and RCF3 regulate overlapping pathways, but they show alternating epistasis in different pathways. Therefore, the RCF3-CPL1 complex likely has multiple mode of function.

## Discussion

Arabidopsis CPL1 has been identified as a negative regulator of osmotic stress responses. The importance of the C-terminal region containing the dsRBM has been suggested by the observation that the *cpl1-2* mutation, which introduces a premature stop codon upstream of the dsRBM1, causes a strong phenotype in osmotic stress-responsive gene expression [3], [5]. However, the role of dsRBMs had not been established, as they are not necessary for catalytic activity of CPL1 [5], and no dsRNA binding has been reported for CPL1. Here we report that CPL1 functionally interacts with RCF3, a KH domain protein, via dsRBM1. KH domains of RCF3 show sequence similarity to those in hnRNP K and PCBP family proteins. The hnRNP K and PCBP KH domains can bind to single-stranded DNA/RNA and function in diverse cellular processes, such as transcriptional regulation [20], [21], mRNA stability [50], and translational control [17]. The target sequences include promoter cis-elements [20], [21], mRNA [50], pre-mRNA [19], and C-rich DNA/RNA sequences such as telomere repeats [51] and viral RNA [52], [53]. Nucleotide binding by the KH domain occurs in a cleft structure formed by α1 and α2 helixes and the GXXG motif in between [54], [55]. The GXXG motif enables formation of a sharp turn structure between α1 and α2, and also can interact with phosphate backbone of the bound oligonucleotide. Of the five KH domains in RCF3, KH2 has an extra single amino acid insertion in the GXXG motif and KH5 entirely lacks the GXXG motif. Such sequence deviation can substantially change conformation, particularly of KH5, which may no longer have the typical KH domain conformation.

Genetic complementation analyses established that only dsRBM1, which is required for interaction with RCF3, is essential in the CPL1 C-terminal region. Currently, dsRNA binding activity of CPL1 has not been established, but dsRBM often functions as a protein-protein interaction module rather than a dsRNA binding site for various proteins [56]. Therefore, it is plausible that the role of the CPL1 C-terminus is to tether the phosphatase domain of CPL1 to RCF3 via dsRBM1 and target the complex to speckles. Speckles are enriched for pre-mRNA splicing machinery and partially overlap with sites of active transcription [57]. Since different types of speckles associated with distinct sets of splicing factors exist [58], predicting the function of the speckle-localized CPL1-RCF3 complex will require further knowledge of speckle proteins and RNAs residing with CPL1-RCF3.

It should be noted that KH domains can bind to single stranded DNA or RNA; therefore, RCF3 could target CPL1 to the promoter of a target gene, and/or the nascent mRNA where CPL1 may dephosphorylate pol II CTD Ser5-PO_4_. Since phosphorylation of Ser5 is required for early stages of transcription, such as promoter escape and mRNA capping [59], [60], tethering CPL1 to target genes/transcripts can facilitate the repressor function of CPL1. Hence, RCF3 may provide specificity to CPL1 by binding to the target DNA/RNA. Binding of the CPL1-RCF3 complex to the target sequence may be achieved by unoccupied KH1, 2, and 5 of RCF3, or by KH3 and KH4 in cooperation with associated dsRBM1 of CPL1. Since KH domains typically recognize 4-base sequences, RCF3 potentially binds to a broad range of targets. In addition, the predicted DNA/RNA binding interface of RCF3, i.e., α1 and to a lesser extent α2 helixes, shows a high level of sequence diversity among the five RCF3 KH domains, suggesting a broad specificity of RCF3 in target recognition. This may explain why CPL1/RCF3 can regulate gene expression in diverse signalling systems such as osmotic stress/ABA [2], [3], wounding [61], heat [16], and Fe deficiency signalling [1].

CPL1 and RCF3 regulate similar sets of genes, suggesting their coordinated function in abiotic stress responses. However, while both *cpl1-6* and *rcf3-2* enhanced the transcriptional response to Fe-deficiency, *rcf3-2* repressed the response to cold stress, opposite to *cpl1-6*. Moreover, *rcf3-2* was epistatic to *cpl1-6* in cold response, whereas *cpl1-6* was epistatic to *rcf3-2* in Fe-deficiency response. Considering specific localization of CPL1-RCF3 complex (Figure 2) but not individual proteins [5], [16] in nuclear speckles, it is likely that function of RCF3 is to promote CPL1 localization in a certain type of nuclear speckles. Based on this model, repression of Fe-deficiency response requires localization of CPL1 in particular type of nuclear speckles, whereas repression of cold-inducible gene expression requires the CPL1 function outside of the speckles. CPL1 released from RCF3 during the cold stress may interact with alternative partners, such as one or more of 26 isoform of RCF3 encoded by the *Arabidopsis thaliana* genome [22], of which only a few have been characterized. Alternatively, CPL1 may form a complex with miRNA producing machinery SE and HYL1 in dicing bodies [62], [63]. Further studies are necessary to delineate the interaction network and functional specification of CPL1 and its interaction partners in various biological processes.

## Supporting Information

Figure S1 **Identification of the RCF3 as CPL1 interacting protein by mass spectrometry analysis.** (A) Sequenced peptides from CPL1 (gi|62321227). Total Mascot score 342 (B) MS–MS spectrum of the 1593.860 Da peptide. MS–MS spectrum of the 1593.860 Da peptide predicts the amino acid sequence of VEYKPSLVSSTDLR. (C) Sequenced peptides from RCF3 (gi|30696273). Total Mascot score 68 (D) MS–MS spectrum of the 2253.218 Da peptide. MS–MS spectrum of the 2253.218 Da peptide predicts the amino acid sequence of VVGESQGIIDLLQNEIGVDVR.
(TIF)

Figure S2 **Sequence alignment of the five KH domains of RCF3 with typical KH domain proteins.** Five KH domains of RCF3 were aligned with KH domains of hnRNP K and PCBP3 using ClustalW. Conserved amino acids are highlighted in black (identity) or gray (similarity). The conserved GXXG loop sequences are boxed. α1–α3 represent α-helix structures and β1–β3 represent β-sheet structures. hnRNP K, heterogeneous nuclear ribonucleoprotein K (*Homo sapiens*, GenBank Accession No. P61978); PCBP3, poly(rC) binding protein 3 (*Homo sapiens*, GenBank Accession No. AAH12061).
(TIF)

Figure S3 **CPL2 dsRBM does not interact with RCF3.** Growth of PJ69-4A co-transformed with GAL4-AD fused with RanBPM (AD:RanBPM) or RCF3 (AD:RCF3) and GAL4-BD fused with CPL2 dsRBM (BD:C2^602–770^) or full-length CPL1 (BD:CPL1). AD:Ran-BPM was a known Gal4-BD-interacting protein and was used to detect presence of a functional BD:CPL2 bait protein. Cells were grown on synthetic dropout (SD) media without uracil and leucine (-UL) or SD medium without uracil, leucine, histidine and adenine (-ULHA). 2×10^5^ cells were used for (1) and diluted 10-fold for (1/10). Photographs were taken after incubation at 28°C for 48 hours. ADv and BDv indicate vector controls.
(TIF)

Figure S4 **Fine mapping of the CPL1 C-terminal nuclear localization signal.** Various truncated CPL1 C-terminal peptides fused with GFP were transiently expressed in *Arabidopsis* protoplasts. Fluorescent signals from CPL1-GFP (GFP) and RFP-NLS (RFP), a positive control for nuclear localization, were obtained using standard FITC and rhodamine filter sets three days after transformation. Yellow signals on merged images indicate co-localization of GFP- and RFP- fusion proteins. Bars on the right indicate CPL1 peptide region fused to GFP. Green and grey colors of the bars indicate nuclear and cytosolic localization of resulting GFP-fusion proteins, respectively. Scale bars indicate 10 µm.
(TIF)

Data S1 (TXT)
