# Correction: Regulation of Abiotic Stress Signalling by Arabidopsis C-Terminal Domain Phosphatase-Like 1 Requires Interaction with a K-Homology Domain-Containing Protein

**DOI:** 10.1371/journal.pone.0140735

**Published:** 2015-10-09

**Authors:** In Sil Jeong, Akihito Fukudome, Emre Aksoy, Woo Young Bang, Sewon Kim, Qingmei Guan, Jeong Dong Bahk, Kimberly A. May, William K. Russell, Jianhua Zhu, Hisashi Koiwa

There are errors in Figs 6 and 7, and in the figure captions. Please see the corrected Figs 6 and 7 below.

**Fig 6:**
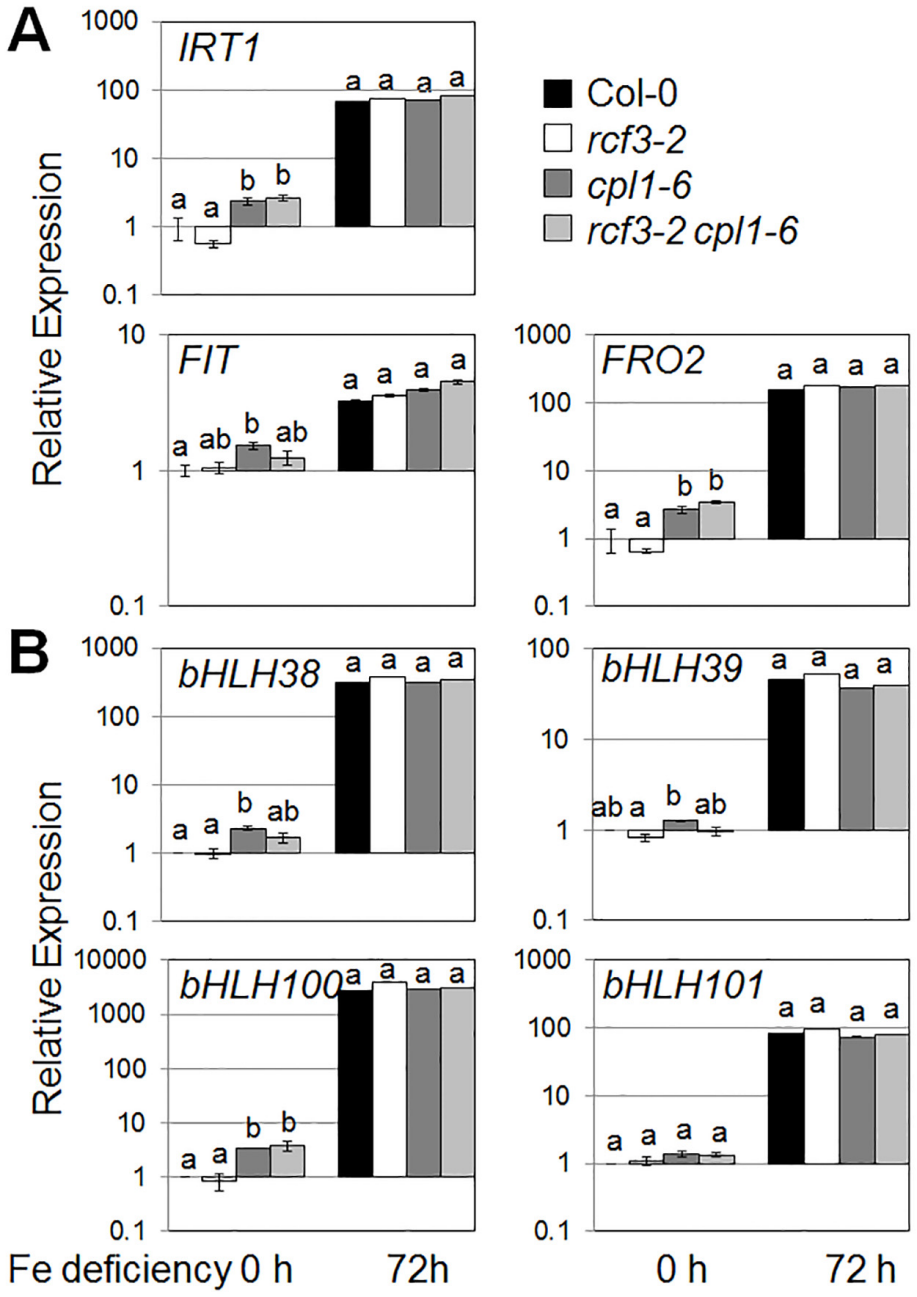
Expression levels of Fe-regulated genes in the roots of Col-0, *rcf3-2*, *cpl1-6*, and *rcf3-2 cpl1-6* under Fe deficiency. (A) Expression levels of FIT-dependent pathway genes. (B) Expression levels of FIT-independent pathway genes. Plants were grown on basal medium containing 100μM Fe-EDTA for 7 days, and then transferred to Fe-deficient medium containing 300 μM ferrozine. Root samples were collected at the time of transfer (0), or 72 h after the transfer. The presented expression levels (relative to untreated Col-0 samples) are mean values of three biological replicates analyzed in duplicates. Bars indicate standard errors of the mean (SEM) of biological replicates. Different letters show significant differences between genotypes under Fe+ and Fe- conditions (p<0.05, one-way ANOVA followed by Tukey’s HSD post hoc test).

**Fig 7:**
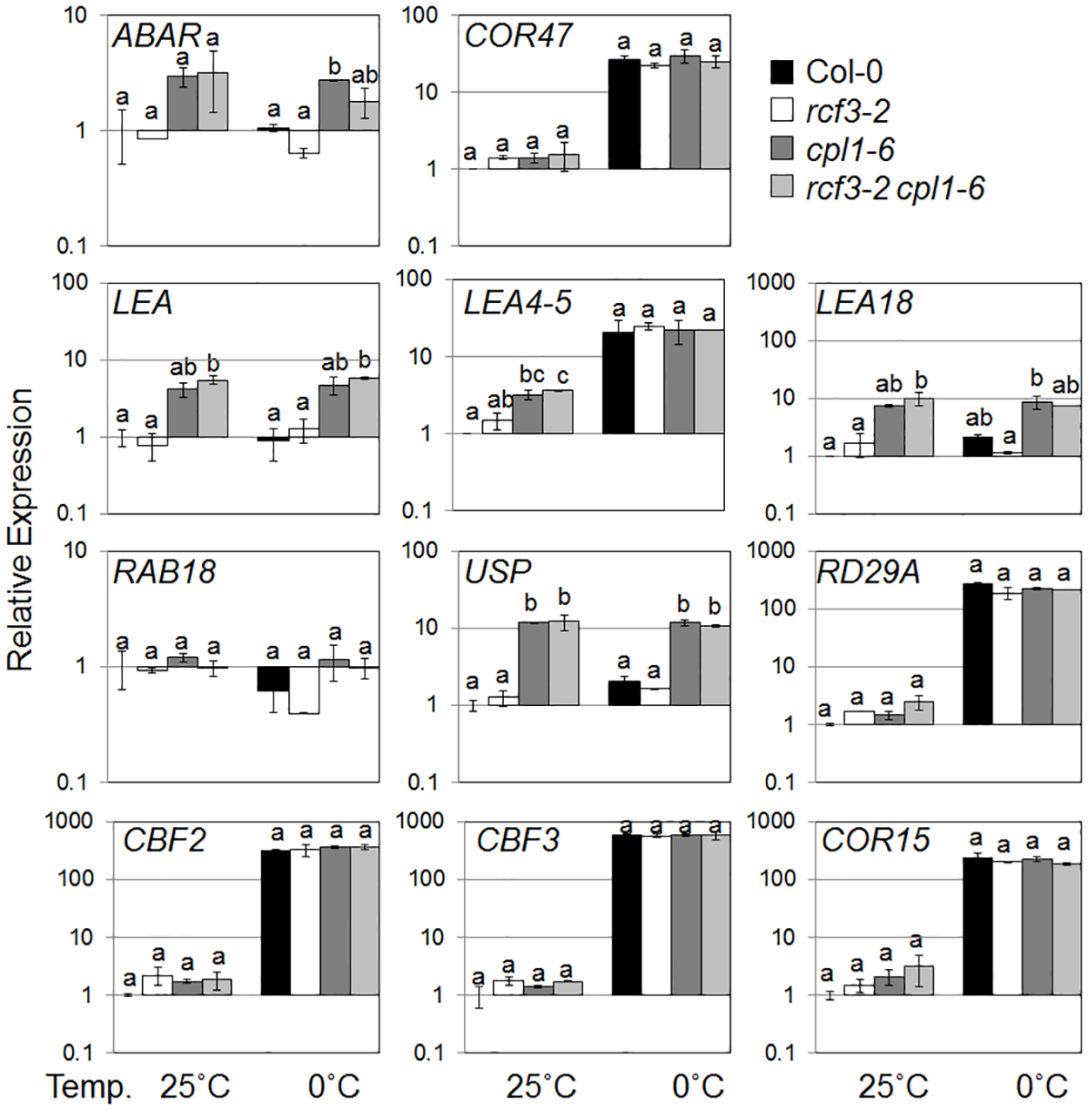
Expression levels of osmotic-stress regulated genes in Col-0, *rcf3-2*, *cpl1-6*, and *rcf3-2 cpl1-6* seedlings. Plants were grown on basal medium for 7 days at 25°C, and then exposed to cold treatment (0°C, 12 h). The presented expression levels (relative to untreated Col-0 samples) are mean values of two biological replicates analyzed in duplicates. Bars indicate standard errors of the mean (SEM) of biological replicates. Different letters show significant differences between genotypes under the same conditions (p<0.05, one-way ANOVA followed by Tukey’s HSD post hoc test).

There are multiple errors in the "CPL1 and RCF3 Function in Overlapping Abiotic Stress Responses" subsection of the Results. Please see the corrected “"CPL1 and RCF3 Function in Overlapping Abiotic Stress Responses" subsection here.

## CPL1 and RCF3 Function in Overlapping Abiotic Stress Responses

Previous data reported in this article were generated using wrong plant materials. In this correction, the plant materials were verified again using PCR, as well as FIT-LUC transgene as phenotypic marker prior to the RT-qPCR analysis. As molecular markers for stress responses, two classes of *CUT*s (*cpl1*-UP Transcripts) that represent various osmotic stress (cold, salinity, etc)-regulated (group I) and Fe-deficiency stress-regulated (group II) genes were used [1]. The analysis of the new data indicated that Fe-responsive transcripts regulated were constitutively upregulated in *cpl1-6* as reported previously [1]. Similar behavior was observed for *LEA* and *USP*, but not other osmotically regulated transcripts tested (Fig 7). In contrast, expression levels of these transcripts in *rcf3-2* did not significantly deviate from Col-0 wild type. The gene expression levels in the *cpl1-6 rcf3-2* double mutant were similar to the *cpl1-6* single mutant. Under Fe deficiency condition, all genotypes produced similar levels of Fe-responsive transcripts. Cold-induction produced two kinds of responses. Transcripts such as *COR47*, *LEA4-5*, *RD29a*, *CBF2/3* and C*OR15* showed strong induction in all genotypes. Only small/moderate induction was observed for *LEA18* and *USP*, for which *cpl1-6* maintained high constitutive expression levels. Transcript levels in *cpl1-6 rcf3-2* were similar to *cpl1-6* single mutant. The new results presented here indicate that co-operation of CPL1 and RCF3 is limited in endogenous Fe and osmotic stress signaling, and CPL1 likely plays a predominant role. However, it is likely that the cooperation does exist in certain targets because *cpl1* and *rcf3* mutants have been co-isolated in multiple forward-genetic screening systems based on stress-inducible gene expression phenotypes.
