# Supplementary figures and images for: Regulation of Abiotic Stress Signalling by Arabidopsis C-Terminal Domain Phosphatase-Like 1 Requires Interaction with a K-Homology Domain-Containing Protein
Source: PLoS One. 2013 Nov 26;8(11):e80509. doi: 10.1371/journal.pone.0080509 (PMC3841200; doi:10.1371/journal.pone.0080509)

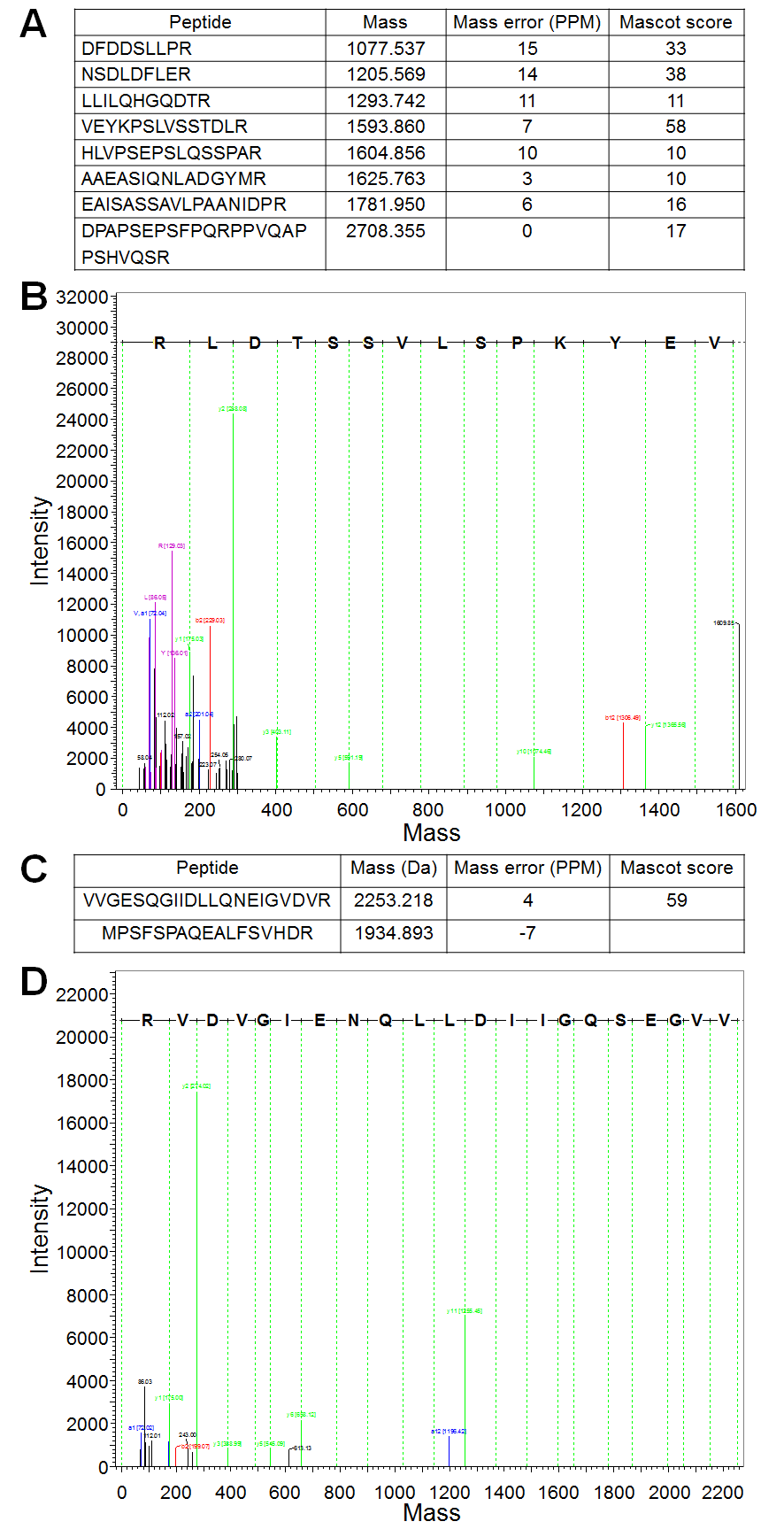

Supplement: Figure S1 — Identification of the RCF3 as CPL1 interacting protein by mass spectrometry analysis. (A) Sequenced peptides from CPL1 (gi|62321227). Total Mascot score 342 (B) MS–MS spectrum of the 1593.860 Da peptide. MS–MS spectrum of the 1593.860 Da peptide predicts the amino acid sequence of VEYKPSLVSSTDLR. (C) Sequenced peptides from RCF3 (gi|30696273). Total Mascot score 68 (D) MS–MS spectrum of the 2253.218 Da peptide. MS–MS spectrum of the 2253.218 Da peptide predicts the amino acid sequence of VVGESQGIIDLLQNEIGVDVR. (TIF) [file pone.0080509.s001.tif]

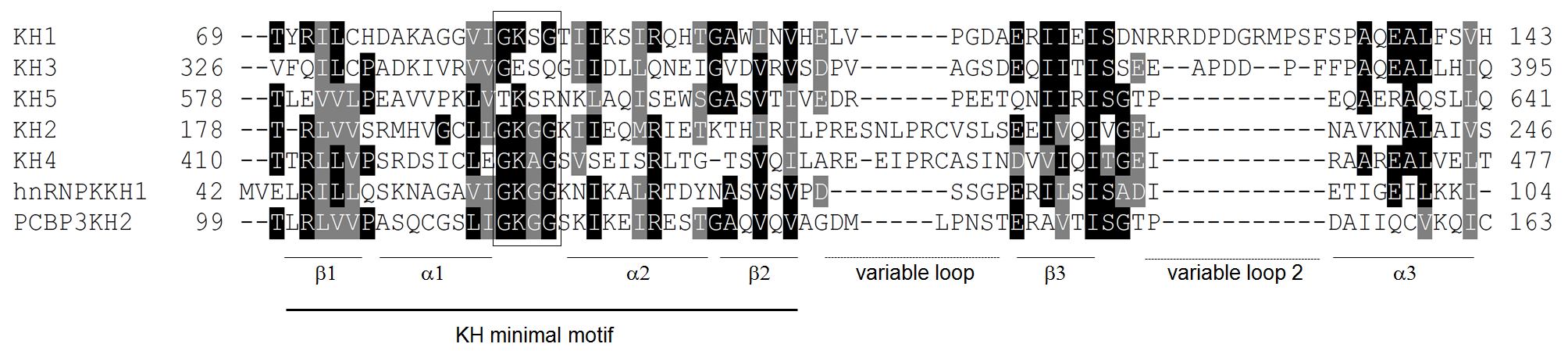

Supplement: Figure S2 — Sequence alignment of the five KH domains of RCF3 with typical KH domain proteins. Five KH domains of RCF3 were aligned with KH domains of hnRNP K and PCBP3 using ClustalW. Conserved amino acids are highlighted in black (identity) or gray (similarity). The conserved GXXG loop sequences are boxed. α1–α3 represent α-helix structures and β1–β3 represent β-sheet structures. hnRNP K, heterogeneous nuclear ribonucleoprotein K (Homo sapiens, GenBank Accession No. P61978); PCBP3, poly(rC) binding protein 3 (Homo sapiens, GenBank Accession No. AAH12061). (TIF) [file pone.0080509.s002.tif]

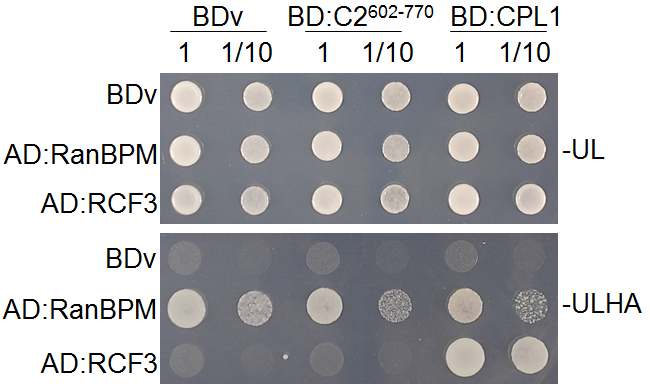

Supplement: Figure S3 — CPL2 dsRBM does not interact with RCF3. Growth of PJ69-4A co-transformed with GAL4-AD fused with RanBPM (AD:RanBPM) or RCF3 (AD:RCF3) and GAL4-BD fused with CPL2 dsRBM (BD:C2602–770) or full-length CPL1 (BD:CPL1). AD:Ran-BPM was a known Gal4-BD-interacting protein and was used to detect presence of a functional BD:CPL2 bait protein. Cells were grown on synthetic dropout (SD) media without uracil and leucine (-UL) or SD medium without uracil, leucine, histidine and adenine (-ULHA). 2×10^5^ cells were used for (1) and diluted 10-fold for (1/10). Photographs were taken after incubation at 28°C for 48 hours. ADv and BDv indicate vector controls. (TIF) [file pone.0080509.s003.tif]

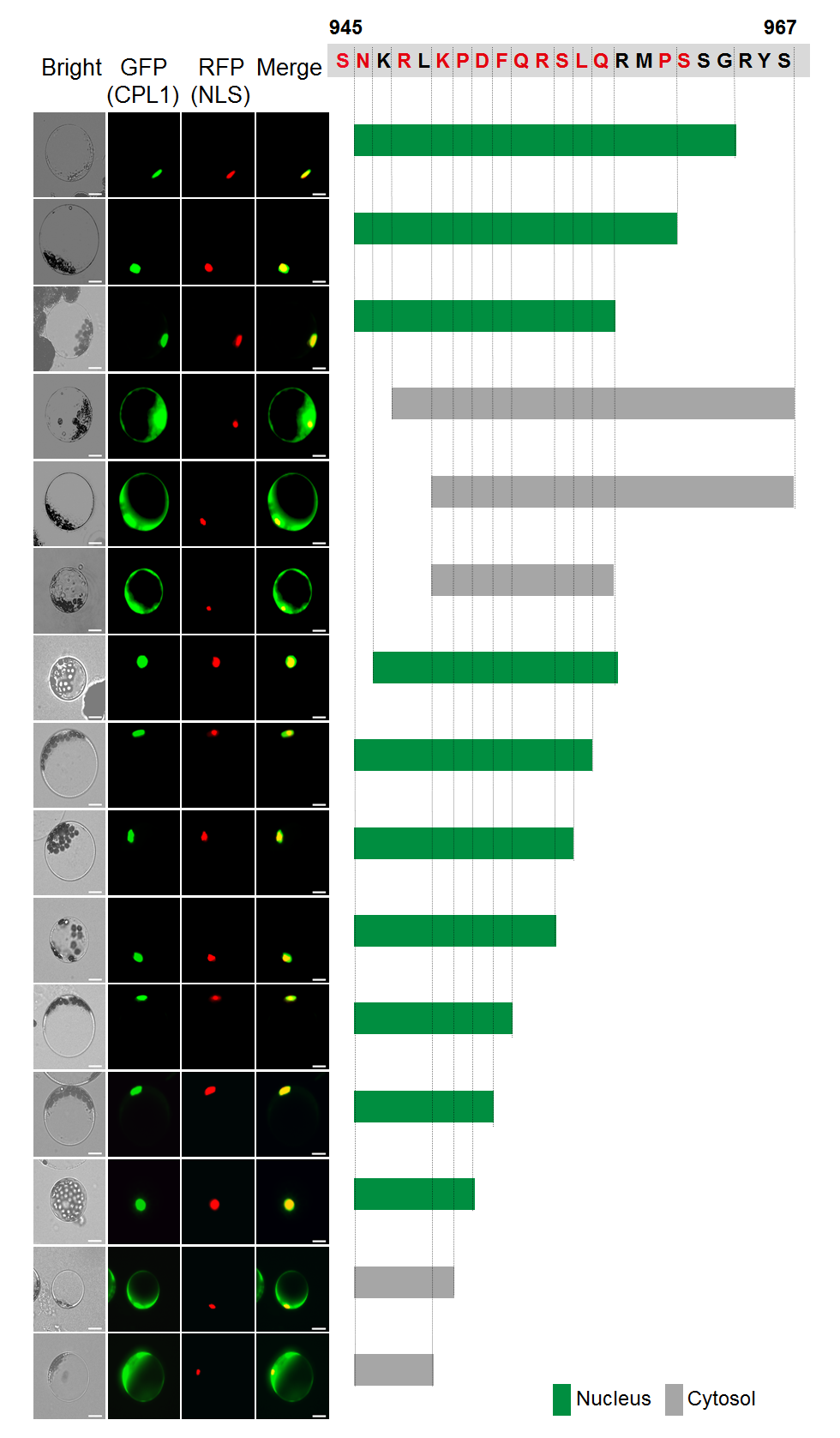

Supplement: Figure S4 — Fine mapping of the CPL1 C-terminal nuclear localization signal. Various truncated CPL1 C-terminal peptides fused with GFP were transiently expressed in Arabidopsis protoplasts. Fluorescent signals from CPL1-GFP (GFP) and RFP-NLS (RFP), a positive control for nuclear localization, were obtained using standard FITC and rhodamine filter sets three days after transformation. Yellow signals on merged images indicate co-localization of GFP- and RFP- fusion proteins. Bars on the right indicate CPL1 peptide region fused to GFP. Green and grey colors of the bars indicate nuclear and cytosolic localization of resulting GFP-fusion proteins, respectively. Scale bars indicate 10 µm. (TIF) [file pone.0080509.s004.tif]
